# Effects of intensity-modulated radiotherapy and chemoradiotherapy on attention in patients with nasopharyngeal cancer

**DOI:** 10.18632/oncotarget.19543

**Published:** 2017-07-25

**Authors:** Qing Wei, Ling Li, Xiao-Dong Zhu, Ling Qin, Yan-Lin Mo, Zheng-You Liang, Jia-Li Deng, Su-Ping Tao

**Affiliations:** ^1^ Department of Radiation Oncology, Cancer Hospital of Guangxi Medical University, Cancer Institute of Guangxi Zhuang Autonomous Region, Nanning 530021, China; ^2^ Department of Radiation Oncology, Yantai Yuhuangding Hospital, Yantai 264000, China; ^3^ Center for Sleep and Cognition, People's Hospital of Guangxi Zhuang Autonomous Region, Nanning 530021, China; ^4^ National Hospital of Guangxi Zhuang Autonomous Region, Nanning 530021, China; ^5^ Department of Computer, Guangxi University, Nanning 530021, China

**Keywords:** nasopharyngeal carcinoma, intensity-modulated radiotherapy, chemoradiotherapy, attention, integrated visual and auditory continuous performance test

## Abstract

This study evaluated the short-term effects of intensity-modulated radiotherapy (IMRT) and cisplatin concurrent chemo-radiotherapy (CCRT) on attention in patients with nasopharyngeal cancer (NPC). Timely detection and early prevention of cognitive decline are important in cancer patients, because long-term cognitive effects may be permanent and irreversible. Thirty-eight NPC patients treated with IMRT (17/38) or CCRT (21/38) and 38 healthy controls were recruited for the study. Neuropsychological tests were administered to each patient before treatment initiation and within a week after treatment completion. Changes in attention performance over time were evaluated using difference values (D-values). Decreased attention was already observable in patients with NPC prior to treatment. Baseline quotient scores for auditory attention, auditory and visual vigilance, and auditory speed were lower in patients treated with CCRT than in healthy controls (P=0.037, P=0.001, P=0.007, P=0.032, respectively). Auditory stamina D-values were higher in patients treated with IMRT alone (*P*=0.042), while full-scale response control quotient D-values were lower in patients treated with CCRT (*P*=0.030) than in healthy controls. Gender, depression, education, and sleep quality were each related to decreased attention and response control. Our results showed that IMRT had no negative acute effects on attention in NPC patients, while CCRT decreased response control.

## INTRODUCTION

Radiotherapy remains the primary treatment option for nasopharyngeal cancer (NPC), which is prevalent in southern China with an incidence of 25–50 per 100,000 people [1]. With intensity-modulated radiotherapy (IMRT) and effective platinum-based concurrent chemotherapy, NPC patients have achieved excellent long-term outcomes [2, 3]. Thus, patient quality of life post treatment has gained much more focus. There are many short- and long-term side effects of radiotherapy and chemotherapy, such as nausea, vomiting, cognitive dysfunction, xerostomia, dysphagia, and olfactory dysfunction [4]. One of the most common side effects is cognitive dysfunction, which negatively impacts academic achievement [5],career, and quality of life [6–9]. While not all cognitive function domains have been shown to undergo changes, attention decline is one of the most common ailments [10–13]. Attention is inextricably linked to intellectual function and is an essential component of human cognitive function, including perception, executive function, learning, and memory. An improved understanding of attention deficit will be important for improving outcomes for patients undergoing treatment for cancer [14, 15].

RT-induced attention deficits have been recognized in brain tumor cases, especially in children and young adults [13, 16–19]. However, while some investigations of RT-induced cognitive dysfunction in NPC patients concluded that RT negatively affects attention [12, 20], other studies demonstrated opposing results [21, 22]. These discrepancies may have resulted from the use of different neuropsychological tests. Tests used in previous reports had limited sensitivities or systematic abilities to evaluate attention changes in NPC patients, and few groups investigated the different aspects of attention [12, 21]. Additionally, most of these studies examined patients treated with conventional two-dimensional radiotherapy (2D-RT)[12, 20, 21]. However, routinely-used intensity-modulated radiation therapy (IMRT) permits the application of higher radiation doses to the tumor without significant radiation-associated toxicities [23], potentially alleviating RT-induced attention decline. Most research examining the cognitive effects of RT in NPC patients has concentrated on long-term neurocognitive sequelae [12, 20–22, 24], but few studies have described the short-term effects of IMRT on attention in these patients using a systematic tool. Although we previously reported no acute attention decline in NPC patients treated with IMRT [25], our analysis lacked a healthy control group, and it was difficult to determine baseline cognitive functions.

Many studies have shown that chemotherapy can induce cognitive decline in patients, especially in women undergoing treatment for breast cancer [6, 26]. Attention problems of varying severity during and after chemotherapy are commonly observed [7, 10, 11]. However, whether platinum-based concurrent chemotherapy increases risk of attention deficit in NPC patients treated with IMRT has not been described.

This prospective study evaluated attention performance in NPC patients before and within one week after completion of cisplatin concurrent chemo-radiotherapy (CCRT) or IMRT alone, as compared with a healthy control group. We assessed attention using the integrated visual and auditory continuous performance test (IVA/CPT). We also explored potential factors influencing attention in NPC patients.

## RESULTS

Thirty-eight patients with NPC and 38 healthy control subjects were assessed in this study (Table 1). Mean patient age (26 males and 12 females) was 43.3 years (range, 23–62 years). Mean healthy control age (23 males and 15 females) was 40.3 years (range, 25–63 years). Age, gender, and education level did not differ significantly between two groups. Among the patients, 17 completed the IMRT alone (IMRT alone subgroup), and 21 received one to three cycles of concurrent cisplatin chemotherapy (CCRT subgroup). In the CCRT subgroup, one patient received one cycle, four patients received two cycles, and 16 patients received three cycles. Age, gender, and education level did not differ significantly within the IMRT alone subgroup, CCRT subgroup, and healthy controls. However, NPC staging, and doses to whole brain and the temporal lobes differed between the IMRT alone and CCRT subgroups. No radionecrosis was identified in the 38 patients according to cranial MRI. Sixteen patients (13 from the IMRT alone subgroup and three from the CCRT subgroup) showed complete remission (CR) and 22(four from the IMRT alone subgroup and 18 from the CCRT subgroup) showed partial remission (PR) within one week after treatment completion. None of the patients progressed during treatment.

**Table 1:** Characteristic of patients and healthy controls

Variable	IMRT alone (n=17)	CCRT (n=21)	Healthy controls (n=38)	P
**Age(years) (M**±SD)	43.5±10.4	43.2±11.9	40.3±10.2	0.479
**Gender**				0.886
Female (%)	11(64.7%)	14(66.7%)	23(60.5%)	
Male (%)	6(35.3%)	7(33.3%)	15(39.5%)	
**Education (years) (M±SD)**	10.1±4.5	11.1±4.2	10.6±4.2	0.773
**NPC staging (7th UICC)**				0.002
Stage I (%)	1(5.9%)	0(0.0%)		
Stage II (%)	12(70.6%)	3(14.3%)		
Stage III (%)	3(17.6%)	12(57.1%)		
Stage IV (%)	1(5.9%)	6(28.6%)		
**Mean dose (Gy) to whole brain (M**±SD)	7.75±1.09	9.56±1.69		0.000
**Maximal dose (Gy) to whole brain (Max/Med/Min)**	77.73/66.33/59.28	85.16/77.80/62.46		0.001
**Mean dose (Gy) to temporal lobes (M**±SD)	14.30±3.40	17.22±3.17		0.010
**Maximal dose (Gy) to temporal lobes (Max/Med/Min)**	79.29/66.75/58.23	89.75/77.72/63.73		0.002
**V60 of temporal lobes (%) (Max/Med/Min)**	4.39/0.63/0.00	7.73/3.05/0.34		0.006

IMRT, intensity-modulated radiotherapy; CCRT, concurrent chemoradiotherapy; M, mean; SD, standard deviation; NPC, nasopharyngeal cancer; UICC, = Union for International Cancer Control; Max, maximum; Med, median; Min, minimum; V60, percentage of temporal lobe volume that received more than 60 Gy.

### Baseline attention performance

Baseline quotient scores for vigilance (omissions) and speed (reaction time), both auditory and visual, were lower in NPC patients (*n*=38) than in healthy controls (*n*=38) (105 /15.8 vs.106/1.0, z = −3.330, *P* = 0.001; 103/4.3 vs. 105/2.3, z = −3.090, *P* = 0.002; 97.1±15 vs. 105.4±18.6, t (74) = −2.120,*P* = 0.037; 98.0/19.5 vs. 104.0/11.3, z = −2.319, *P* = 0.020). Other quotient scores and full scores for IVA-CPT did not differ between the two groups (Table 2).

**Table 2:** IVA/CPT scores at baseline between two groups

CPT scores		Patients (*n*=38)	Healthy controls (*n*=38)	t(74)/z*	P
FSRCQ (M±SD)		99.8±16.1	100.7±15.3	−0.234	0.816
ARCQ (M±SD)		97.6±17.0	98.7±14.9	−0.287	0.775
VRCQ *(Median/IQR)		102.5/9.3	104.0/6.3	−0.286	0.775
FSAQ *(Median/IQR)		94.5/25.3	102.5/20.8	−1.741	0.082
AAQ (M±SD)		96.3±16.9	103.2±14.4	−1.912	0.060
VAQ *(Median/IQR)		93.0/23.8	97.0/24.8	−1.528	0.126
Prudence*	Auditory (Median/IQR)	109.0/13.5	107.0/9.3	−1.140	0.254
	Visual (Median/IQR)	113.0/14.3	109.0/3.0	−0.825	0.410
Consistency	Auditory (M±SD)	91.8±18.7	90.3±15.6	0.360	0.720
	Visual* (Median/IQR)	94.5/18.8	100.0/17.5	−1.408	0.159
Stamina	Auditory (M±SD)	99.7±16.8	103.0 ±17.8	−0.850	0.398
	Visual (M±SD)	100.1±13.2	100.2±15.1	−0.049	0.961
Vigilance*	Auditory (Median/IQR)	105.0/15.8	106.0/1.0	−3.330	0.001
	Visual (Median/IQR)	103.0/4.3	105.0/2.3	−3.090	0.002
Focus	Auditory (M±SD)	99.7±12.7	96.5±13.5	1.052	0.296
	Visual (M±SD)	98.1±18.4	91.6±17.5	1.557	0.124
Speed	Auditory(M±SD)	97.1±15.0	105.4±18.6	−2.120	0.037
	Visual* (median/IQR)	98.0/19.5	104.0/11.3	−2.319	0.020

M, mean; SD, standard deviation; IQR, interquartile range; FSRCQ, full scale response control quotient; ARCQ, auditory response control quotient; VRCQ, visual response control quotient; FSAQ, full scale attention quotient; AAQ, auditory attention quotient; VAQ, visual attention quotient; “*”: the t-test was used for normal distributed variables while Mann-Whitney U test was used for the non-normal distributed variables.

A pairwise comparison revealed that auditory attention quotient (AAQ), auditory and visual vigilance quotient, and auditory speed quotient were lower in patients treated with CCRT compared to healthy controls (P=0.037, P=0.001, P=0.007, P=0.032, respectively) (Table 3). There were no differences among patients treated with IMRT alone. Auditory speed quotient in patients treated with CCRT was also lower than that in IMRT patients (101.7 ± 14.5 vs.93.4 ± 14.7, P = 0.045). Other IVA-CPT scores showed no differences between the IMRT and CCRT subgroups at baseline.

**Table 3:** IVA/CPT scores at baseline among three groups

CPT scores (M±SD)		IMRT alone (*n*=17)	CCRT (*n*=21)	Healthy controls (*n*=38)	F (2.73)/X^2^(2,N=76)	P
FSRCQ		96.9±17.2	102.2±15.3	100.7±15.3	0.574	0.566
ARCQ		96.5±16.6	98.6±17.7	98.7±14.9	0.121	0.886
VRCQ		98.7±16.1	104.4±12.0	102.1±14.3	0.793	0.457
FSAQ		96.9±16.1	91.3±15.9	102.0±17.6	2.780	0.069
AAQ		101.2±15.6	92.4±17.3	103.2±14.4	3.371	0.040*
VAQ		93.1±17.4	89.4±17.3	99.5±18.1	2.347	0.103
Prudence	Auditory	103.6±13.2	104.6±13.4	103.3±10.4	0.107	0.898
	Visual	103.1±15.2	110.5±7.8	105.8±12.6	1.846	0.165
Consistency	Auditory	92.9±18.5	90.9±19.3	90.3±15.6	0.128	0.880
	Visual	90.2±22.7	97.9±13.1	99.0±16.4	1.527	0.214
Stamina	Auditory	97.1±17.0	101.8±16.7	103.0 ±17.8	0.706	0.497
	Visual	100.8±13.5	99.5±13.3	100.2±15.1	0.039	0.961
Vigilance	Auditory (Max/Med/Min)	109/106/45	107/105/53	113/106/80	11.876	0.003*
Visual	(Max/Med/Min)	106/103/76	106/103/43	115/105/69	9.621	0.008*
Focus	Auditory	101.3±13.4	98.3±12.2	96.5±13.5	0.788	0.458
	Visual	97.4±19.5	98.6±17.9	91.6±17.5	1.219	0.301
Speed	Auditory	101.7±14.5	93.4±14.7	105.4±18.6	3.429	0.038*
	Visual	96.5±15.5	91.5±19.6	102.8±16.7	2.993	0.056

M, mean; SD, standard deviation; FSRCQ, full scale response control quotient; ARCQ, auditory response control quotient; VRCQ, visual response control quotient; FSAQ, full scale attention quotient; AAQ, auditory attention quotient; VAQ, visual attention quotient; Max, maximum; Med, median; Min = minimum. *: Results of past hoc pairwise comparison: AAQ: P (IMRT vs. CCRT) = 0.264, P (IMRT vs. healthy controls) > 0.999, P (CCRT vs. healthy controls) = 0.037; Audirory speed: P (IMRT vs. CCRT) = 0.045, P (IMRT vs. healthy controls) > 0.999, P (CCRT vs. healthy controls) = 0.032; auditory vigilance: P (IMRT vs. CCRT) = 0.383, P (IMRT vs. healthy controls) = 0.031, P (CCRT vs. healthy controls) = 0.001; visual vigilance: P (IMRT vs. CCRT) = 0.706, P (IMRT vs. healthy controls) = 0.019, P (CCRT vs. healthy controls) = 0.007.

### IVA/CPT score changes over time

**Figure 1:**
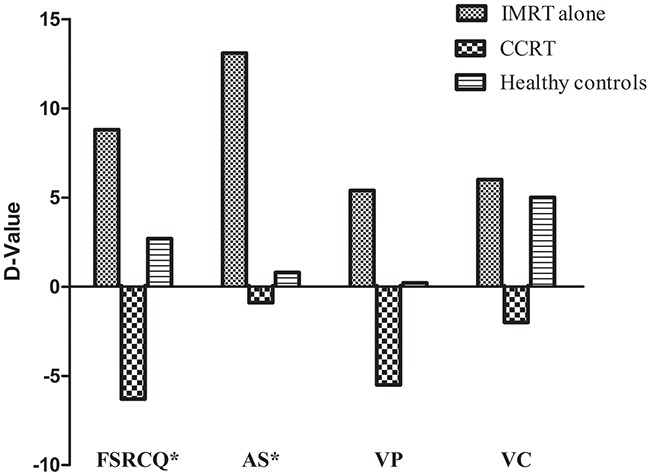
Selected D-values of IVA/CPT illustrating pre-treatment results subtracted from post-treatment results

D-values for each IVA-CPT score did not differ between NPC patients (*n*=38) and healthy controls (*n*=38) (*P*>0.05). However, a comparison of the three groups revealed differences in FSRCQ, visual prudence (commissions) (VP), visual consistency (intra-individual variability) (VC), and auditory stamina (fatigue) (AS) D-values (*P*=0.001, *P*=0.014, *P*=0.030, and *P*=0.034, respectively). FSRCQ, VP, and VC were lower in CCRT patients than those treated with IMRT alone, but these differences were not significant except FSRCQ when compared with demographically matched healthy controls. Auditory stamina (fatigue) D-values were higher in patients treated with IMRT alone (13.1±22.7 vs. 0.8±15.5, *P*=0.042), while FSRCQ D-values were lower in CCRT patients compared to healthy controls (-6.3±13.1 vs. 2.7 ±11.7, *P* = 0.030) (Figure 1). A three-group repeated measures ANOVA indicated a difference in AAQ among the three groups (F (2, 73) =3.771, P=0.028), but there was no significant AAQ change over time (F (1, 73) =1.350, P=0.249) (Table 4). Follow-up comparisons indicated that AAQ was lower in CCRT patients than in healthy controls at baseline (92.43±17.35 vs. 103.24±14.39, P= 0.037). Mean auditory consistency (intra-individual variability) changed over time (F (1, 73) =10.894, P=0.001), and mean auditory consistency scores at follow-up were higher than at baseline. However, there were no significant differences among groups.

**Table 4:** Repeated measures ANOVA for AAQ among three groups

Effect	df	F	Significance
ARCQ			
Between Group	2,73	0.374	0.690
Time	1,73	1.492	0.226
Group×Time	2,73	3.207	0.046
VRCQ			
Between Group	2,73	0.109	0.897
Time	1,73	0.824	0.367
Group×Time	2,73	4.808	0.011
AAQ			
Between Group	2,73	3.771	0.028*
Time	1,73	1.350	0.249
Group×Time	2,73	0.644	0.528
Auditory consistency			
Between Group	2,73	0.420	0.659
Time	1,73	10.894	0.001
Group×Time	2,73	0.573	0.566
Visual stamina			
Between Group	2,73	0.363	0.697
Time	1,73	1.036	0.312
Group×Time	2,73	0.148	0.862
Auditory focus			
Between Group	2,73	0.571	0.568
Time	1,73	0.083	0.774
Group×Time	2,73	0.776	0.464

Note: ARCQ, auditory response control quotient; VRCQ, visual response control quotient; AAQ, auditory attention quotient. *: The auditory attention quotient of patients in CCRT group were significantly lower than healthy controls according to the post hoc Bonferroni test (P=0.023).

### Psychological characteristics and sleep quality

At baseline, NPC patient sleep disorder incidence was higher than in healthy controls (28.9% vs.7.9%, P = 0.018). However, there were no differences in depression and anxiety incidence between the two groups (7.9% vs. 2.6%, P = 0.607; 10.5 vs. 0.0%, P = 0.123). A three-group comparison revealed differences in anxiety and sleep disorder incidence (X^2^ (2) =5.214, P=0.036; X^2^ (2) =6.3, P=0.041, respectively), although post hoc pairwise comparisons indicated no differences.

During follow-up, depression, anxiety, and sleep disorder incidences in patients treated with CCRT, and depression and sleep disorder incidences in patients treated with IMRT were higher than in healthy controls (X^2^ (1) = 20.330, P = 0.000; X^2^ (1) = 7.054, P = 0.008; X^2^ (1) = 18.265, P = 0.000; X^2^ (1) = 8.532, P = 0.003; X^2^ (1) = 13.160, P = 0.000, respectively). Incidences of depression, anxiety, and sleep disorder were the same between the IMRT and CCRT subgroups. Depression and sleep disorder incidences in both the IMRT and CCRT subgroups increased compared to baseline (35.3% vs. 0%, X^2^(1) = 5.060, P = 0.024; 57.1% vs. 14.3%, X^2^ (1) = 8.4, P = 0.004; 64.7% vs. 23.5%, X^2^ (1) = 5.846, P = 0.016; 71.4% vs. 33.3%; X^2^(1) = 6.109, P = 0.013, respectively). (Table 5).

**Table 5:** Depression, anxiety, and sleep quality among three groups

	Baseline				Follow-up					
	IMRT alone	CCRT	Healthy controls	P *	IMRT alone	CCRT	Healthy controls	P **	P***	P****
**Morbidit of depression**				0.115				0.000	0.024	0.004
Depression (%)	0(0.0)	3(14.3)	1(2.6%)		6(35.3)	12(57.1)	1(2.6%)			
No depression (%)	17(100)	18(85.7)	37(97.4%)		11(64.7)	9(42.9)	37(97.4%)			
**Morbidity of anxiety**				0.036				0.003	0.595	0.432
Anxiety (%)	1(5.9)	3(14.3)	0(0.0%)		3(17.6)	5(23.8)	0(0.0%)			
No anxiety (%)	16(94.1)	18(85.7)	38(100%)		14(82.4)	16(76.2)	38(100%)			
**Sleep quality**				0.041				0.000	0.016	0.013
Poor sleep quality (%)	4(23.5)	7(33.3)	3(7.9%)		11(64.7)	15(71.4)	6(15.8%)			
Good sleep quality (%)	13(76.5)	14(66.7)	35(92.1%)		6(35.3)	6(28.6)	32(84.2%)			

Notes: IMRT, intensity-modulated radiotherapy; CCRT, concurrent chemoradiotherapy; “*”: Depression, anxiety, and sleep quality among three groups at baseline; “**”: Depression, anxiety, and sleep quality among three groups at follow up; “***”: Depression, anxiety and sleep of NPC patients with IMRT alone before and after radiotherapy; “****”: Depression, anxiety and sleep of NPC patients with CCRT before and after treatment.

### Correlations

Variables such as age, gender, educational background, clinical stage, pre-RT SDS scores, pre-RT SAS scores, and pre-RT PSQI scores were investigated as potential attention influencing factors in pre-treatment patients of the CCRT subgroup. Additionally, concurrent chemotherapy cycle, whole brain and temporal lobes dosage, post-treatment SDS scores, post-treatment SAS scores, and post-treatment PSQI scores were investigated as potential factors decreasing FSRCQ in patients treated with CCRT. We found that pre-treatment SDS scores and pre-treatment auditory speed were negatively correlated (correlation coefficient r = −0.484, P = 0.026), and pre-RT visual vigilance was correlated with gender (males had a relatively lower visual vigilance before treatment than females) (r_s_= −0.512, P = 0.018). None of the variables examined correlated with pre-treatment auditory attention or pre-treatment auditory vigilance. FSRCQ D-value was correlated with educational background and post-treatment PSQI scores (r = 0.434, P = 0.049; r = −0.479, P = 0.028). However, the concurrent chemotherapy cycle, and the doses to whole brain and temporal lobes were not related to decreased FSRCQ in patients treated with CCRT.

## DISCUSSION

To our knowledge, this is the first prospective study to investigate the effects of IMRT and CCRT on attention in NPC patients compared with healthy controls using a standard, comprehensive test. To explore potential factors affecting attention, we evaluated NPC patient mood and sleep quality before and after treatment. Compared with healthy controls, patients treated with CCRT demonstrated poorer pre-treatment performance in several aspects of attention, and their response control performances decreased post-treatment. However, the auditory stamina of patients treated with IMRT alone improved post-RT. IMRT and CCRT could increase incidences of depression and sleep disorders. Patients who presented depression pre-RT had an increased likelihood of slower baseline auditory speeds. Males were found to have lower visual vigilance pre-RT than females. Finally, patients with sleep disorders post-treatment and lower education levels were more likely to exhibit decreased FSRCQ.

IMRT and platinum-based concurrent chemotherapy have improved survival for locoregionally advanced NPC. However, treatment side effects, such as cognitive decline, can negatively impact patient quality of life. Patients may develop late cognitive impairments in attention, short-term memory, language abilities, list-generating fluency, and executive function years after radiation [12, 21, 22]. Most such impairments occur in patients with cerebral radionecrosis [12, 21]. However, recent studies showed that patients might suffer cognitive decline without cerebral radionecrosis [20, 22]. Because long-term cognitive disorders are not always reversible, monitoring for cognitive decline early after treatment is important. We can prevent long-term nonreversible cognitive disorders by treating early stage short-term cognition decline, and early detection markers are essential for prevention and treatment of even mild cognitive impairment (MCI). Attention is considered especially vulnerable to stress factors, such as chemotherapy and radiotherapy, and is the most impaired at short-term follow-up [27–29]. Attention decline may be an important clinical marker and predictor of early MCI and may help identify persons at increased risk. Thus, our study used an objective, standard test to assess the short-term effects of IMRT and CCRT on attention in patients with NPC.

IVA/CPT is widely used as an objective diagnostic tool for attention deficit hyperactivity disorder (ADHD) in children and adults [30]. It has also been used to evaluate deficits in attention and response control in individuals diagnosed with brain damage, such as stroke and mild traumatic brain injury (mTBI) [31]. Thus, IVA/CPT permits the evaluation of different attention features and facilitates recognition of early changes in attention in patients with NPC.

Our results showed that patients treated with CCRT had poorer performance than healthy controls in terms of auditory attention, auditory speed, and auditory and visual vigilance pre-treatment. CCRT patients also exhibited poorer baseline auditory speeds than patients treated with IMRT, indicating that attention may be associated with the tumor itself. Previous studies showed that patients with non-central nervous system (CNS) tumors could experience cognitive dysfunction prior to any treatment [32–34]. In a study based on a self-reported subjective scale,48% of 595 patients diagnosed with cancer reported concentration problems prior to treatment initiation [35]. Several hypotheses have been proposed to explain this phenomenom, including the biology of cancer itself (the inflammatory response triggering neurotoxic cytokine production) and shared potential risk factors for cancer progression and cognitive dysfunction (defective DNA repair mechanisms associated with both cancer and neurodegenerative disorders) [36, 37]. CCRT subgroup clinical stage was higher than in the IMRT subgroup. Higher tumor burden may be more likely to trigger neurotoxic cytokine production, possibly resulting in attention decline. Patient demographics, psychological burdens, and sleep disorders resulting from the cancer diagnosis may also be related to pretreatment attention abnormalities [38–40]. Many patients suffer anxiety and depression after cancer diagnosis [38, 40], and these psychological problems could lead to attention decline [39]. In our study incidences of depression and anxiety in patients were higher than in healthy controls, although the differences were not significant (7.9% vs. 2.6%, P=0.607; 10.5 vs. 0.0%, P=0.123, respectively). However, pre-treatment SDS scores and pre-treatment auditory speeds were negatively correlated, suggesting that patients with depression had poorer auditory speed performances. Males also exhibited inferior visual vigilance performances pre-treatment compared to females. We also found that patients with sleep disorders and lower education levels were more likely to show decreased FSRCQ post-treatment. Although several attention subscales were worse in NPC patients compared with healthy controls, full-scale attention quotients were similar between the two groups. This indicates that patients with NPC may suffer subtle changes in attention before a decrease in overall attention index is measured.

We observed no decline in attention performance after IMRT alone, and patient auditory stamina improved. Stamina compares patient mean response times during the first 200 trials to the last 200 trials, and is used to identify problems related to sustaining attention and effort over time. We found that patients had improved auditory attention stability in the continuous test after IMRT. This may be attributed to disease control and reduced inflammatory cascade activity following successful treatment. Additionally, lower baseline scores in the IMRT subgroup may allow for a greater improvement range than in healthy controls. However, Hua,*et al*. reported that RT could impair auditory attention/concentration in patients with NPC a median of 1.7 years after conventional radiotherapy completion [20]. This difference may be attributed to IMRT, which spares normal tissues from the target volume. This may also suggest that radiation has delayed and progressive effects, and a long-term decline in attention after IMRT may occur in NPC patients.

CCRT patient response control was similar to that of healthy controls and the IMRT subgoup at baseline, but declined after treatment. Gan,*et al.*[41] found that patients with head and neck cancer (excluding NPC) receiving CCRT had poorer performance in objective attention than patients receiving RT alone, although this difference was not significant(possibly due to small sample size). These findings agreed with our results, and reduced performance in CCRT patients may be due to cisplatin neurotoxicities, including transient cortical blindness, seizures [42, 43], encephalopathy [44], and histologic abnormalities in the brain [45]. CCRT might introduce additional risk, because concurrent radiotherapy can damage the blood-brain barrier and may facilitate cisplatin entry into the CNS [46]. Chemotherapy side effects, including nausea, vomiting, and lack of appetite, may also decrease patient response control. Hsiao,*et al.*[22] reported no attention decline in patients with NPC at least 12 months after IMRT or CCRT, demonstrating that these deficits may improve gradually without additional insult. Almost 30% of patients with NPC suffered poor sleep before treatment initiation, and, in agreement with our previous findings, our results confirmed that depression and sleep disorder incidences increased after treatment [25]. This can likely be attributed to radiation and chemotherapy side effects.

Our study had several limitations. First, our patient/control sample size was small. Second, D-value standard deviations were relatively large, which may have been a consequence of small sample size and fluctuations in mental status and sleep quality, which could affect attention. Third, two patients were excluded from the analysis for failure to attend the follow-up assessment because of severe side effects, which may have influenced the results. Finally, we did not eliminate the effects of treatment toxicity. Thus, the present findings should be interpreted with caution. Still, subtle changes on attention may be markers for cognitive dysfunction. Timely detection and early prevention are important, because long-term cognitive effects may be permanent and irreversible.

In conclusion, our findings suggest that attention declines observed in patients with NPC are present prior to any treatment. IMRT alone had no negative short-term effects on attention in these patients, and auditory stamina performance improved within one week after treatment completion. In contrast, cisplatin concurrent chemotherapy decreased response control in NPC patients. Male patients with depression were more likely to have attention deficits prior to treatment, and patients with sleep disorders or lower education levels were more likely to have decreased response control post-treatment. We suggest that the IVA-CPT be used to measure attention in patients with NPC. Further studies with larger subject populations and longer follow-up times are needed to better delineate the effects of cancer and cancer treatment on attention in patients with NPC.

## MATERIALS AND METHODS

### Patients

We recruited NPC patients between March 2015 and July 2015 through the Department of Radiation Oncology at the Cancer Hospital of Guangxi Medical University. Inclusion criteria were as follows: (1) new pathologic diagnosis of NPC, Union for International Cancer Control (UICC, 7^th^ ed.) stage I to IVb (T1-4, N0-3, M0; T1: nasopharynx and/or oropharynx and/or nasal cavity extension; T2: parapharyngeal extension; T3: bony structure and/or paranasal sinuses extension; T4: intracranial extension and/or cranial nerve, hypopharynx, orbit and/or infratemporal fossa (masticatory space) extension; N0: no regional lymph node metastasis; N1: unilateral metastasis in lymph node(s), uni/bilateral retropharyngeal lymph node, above the supraclavicular fossa, D≤6 cm; N2: bilateral cervical above the supraclavicular fossa, D≤6 cm; N3a: >6 cm in the greatest dimension; N3b: extension to the supraclavicular fossa; M0: no distant metastasis); (2) treated with either IMRT alone or CCRT; (3) aged 18–60 years; (4) no history of CNS disease or malignant disease treated with chemotherapy or radiation; and (5) normal hearing and eyesight. Patients with a history of alcohol/substance abuse, head injury, psychiatric/neuropathic disorder, intellectual disability, and/or those using medications known to affect attention were excluded. Healthy controls meeting the same inclusion (except for the first two) and exclusion criteria were enrolled through their caregivers and hospital staff members. All enrolled participants provided written informed consent. This research was completed in accordance with the Helsinki Declaration and was approved by the Ethics Committee of Guangxi Medical University.

### Intensity-modulated radiotherapy and chemoradiotherapy

All enrolled patients were treated with 6 MV photons using an IMRT technique with nine portals. Patients were immobilized in the supine position with a thermoplastic mask, and the locations of the target and organs at risk were obtained by computed tomography (CT) simulation. CT images were then used to define target volumes and normal tissue structures. The irradiation dose to the primary tumor was 70–72.32 Gy, and 60–70 Gy was applied to positive cervical lymph nodes. All patients were treated with the same fractionation schedule as follows: 1.8–2.26 Gy per fraction, with five daily fractions per week for 6–7 weeks. Temporal lobe and whole brain dose-volume histograms were obtained for all patients. Concurrent chemotherapy consisted of 100 mg/m^2^ cisplatin every three weeks (days 1, 22, and 43 during RT) per cycle to patients with stages III–IVb and some with stage II disease. All patients received a cranial magnetic resonance imaging (MRI) exam before and after treatment. On MRI, gross hypointensity in T1-weighted images, hyperintensity in T2-weighted images, and heterogeneous contrast enhancement in gadolinium-enhanced T1-weighted images on either or both hemispheres indicated edema or necrosis.

### Neuropsychological testing

The IVA/CPT was used to assess patient attention and response control. The test has a high reliability and validity [47, 48], and consists of warm-up, main, and cool-down tests. Subjects were instructed to click the mouse when they saw or heard a “1” (target) and to not click the mouse when they saw or heard a “2” (distractor). Before beginning the test, the system voice was adjusted to a moderate volume to make sure the subjects heard the instructions clearly. The warm-up test makes sure subjects understand the operation rules and prepares them for the main test. Unqualified results in warm-up tests results in an invalid main test score. The main test, lasting approximately 15 min, assesses attention and response control at both auditory and visual levels by measuring omission and commission errors, reaction times, and response variability throughout the test. We selected six global indexes and six primary composite scales to evaluate patient attention deficit characteristics. The following six primary composite scales were assessed for both visual and auditory performance: (1) prudence (commissions) measures impulsivity and response inhibition of non-target stimuli as evidenced by three different types of errors of commission; (2) consistency (intra-individual variability) measures the general reliability and variability of response times, along with the ability to stay on task; (3) stamina (fatigue) identifies problems related to sustaining attention and effort over time; (4) vigilance (omissions) reflects the ability to maintain and direct attention to categorize stimuli as target or non-target and give the appropriate response; (5) focus (intra-individual variability) measures total mental processing speed variability for all correct answers; (6) speed (reaction time) reflects mental processing speed based on the average reaction time for all correct responses throughout the test. The full-scale response control quotient (FSRCQ) is derived from separate auditory and visual response control quotient scores based on visual and auditory prudence, consistency, and stamina scales. The full-scale attention quotient (FSAQ) is based on separate auditory and visual attention quotients derived from equal measures of visual and auditory vigilance, focus, and speed. Quotient scores are presented as standardized scores with a mean of 100 and a standard deviation of 15 based on the normative data from the IVA. Higher scores indicate better performance.

We use the Self-Rating Anxiety Scale (SAS) and Self-Rating Depression Scale (SDS) to assess the subjective symptoms of anxiety and depression, respectively. Both scales consist of 20 items each, and each item is scored on a 1–4 scale based on the answers (the overall assessment is based on total score). After standardization, a cut-off point of ≥50 was used to define anxiety or depression according to the Chinese version of the scales. The SAS and SDS test-retest reliabilities were 0.777 and 0.820 [25], respectively.

The Pittsburgh Sleep Quality Index (PSQI) is a self-rated instrument used to evaluate subjective sleep quality over the month before questionnaire administration. It includes 19 individual self-rated questions that generate 7 component scores. Each component is scored from 0–3, and total score ranges from 0–21. In accordance with the Chinese norm, a total score >5 indicates sleep disturbance, and higher scores indicate poorer sleep quality. The Cronbach's alpha coefficient and test-retest reliability were 0.82–0.83 and 0.85 [49], respectively.

### Patient evaluation procedures

Each patient was evaluated individually in a quiet room using IVA/CPT, SDS, SAS, and PSQI before the initial treatment and within one week after completion of IMRT or CCRT. Healthy controls were subjected to the same assessment over an equivalent time interval (7–8 weeks). Graduate students trained in medicine administered the tests according to standard instructions. The entire assessment took approximately 30–40 minutes per patient.

### Statistical analyses

Sample characteristics and selected item scores were analyzed using standard descriptive statistics. Changes over time in IVA-CPT scores among those with a non-normal distribution were evaluated by the “difference value” (D-value), which was calculated based on the scores obtained at follow-up minus baseline scores. IVA-CPT baseline scores and D-values were compared between groups using the independent samples t-test/one-way analysis of variance (ANOVA) followed by the post hoc Bonferroni pairwise comparison test for variables with a normal distribution. The Mann–Whitney test/Kruskal-Wallis test with post hoc pairwise comparisons using the Mann-Whitney U test was applied for variables with a non-normal distribution. A three-group repeated measures ANOVA was applied for variables that exhibited normal distributions on two tests. Chi square tests were performed to compare the prevalence of depression, anxiety, and poor sleep quality over time and between groups. Correlations between clinical and psychological variables and patient CPT scores were assessed using Pearson or Spearman correlation analyses. All tests were two-sided, and a P<0.05 was considered statistically significant (P<0.017 for the post hoc pairwise comparisons using the Mann-Whitney U test and chi square test). The analysis was performed using the Statistical Package for the Social Sciences (SPSS) version 16.0 (SPSS Inc., Chicago, IL, USA).
